# Progesterone receptor membrane component 1 regulates lipid homeostasis and drives oncogenic signaling resulting in breast cancer progression

**DOI:** 10.1186/s13058-020-01312-8

**Published:** 2020-07-13

**Authors:** Hannah Asperger, Nadia Stamm, Berthold Gierke, Michael Pawlak, Ute Hofmann, Ulrich M. Zanger, Annamaria Marton, Robert L. Katona, Andrea Buhala, Csaba Vizler, Jan-Philipp Cieslik, Eugen Ruckhäberle, Dieter Niederacher, Tanja Fehm, Hans Neubauer, Marina Ludescher

**Affiliations:** 1 Department of Obstetrics and Gynecology, University Hospital and Medical Faculty of the Heinrich-Heine University Duesseldorf, Life Science Center, Merowingerplatz 1A, 40225 Düsseldorf, Germany; 2 NMI TT Pharmaservices, Protein Profiling, Reutlingen, Germany; 3 Dr. Margarete Fischer-Bosch Institute of Clinical Pharmacology and University of Tübingen, Stuttgart, Germany; 4 Institute of Biochemistry, Biological Research Centre, Szeged, Hungary; 5 Institute of Genetics, Biological Research Centre, Szeged, Hungary

**Keywords:** PGRMC1, Breast cancer, Tumor progression, Cholesterol, Lipids, Estrogen receptor α, HER2, EGFR, Breast cancer signaling pathway

## Abstract

**Background:**

PGRMC1 (progesterone receptor membrane component 1) is a highly conserved heme binding protein, which is overexpressed especially in hormone receptor-positive breast cancer and plays an important role in breast carcinogenesis. Nevertheless, little is known about the mechanisms by which PGRMC1 drives tumor progression. The aim of our study was to investigate the involvement of PGRMC1 in cholesterol metabolism to detect new mechanisms by which PGRMC1 can increase lipid metabolism and alter cancer-related signaling pathways leading to breast cancer progression.

**Methods:**

The effect of PGRMC1 overexpression and silencing on cellular proliferation was examined in vitro and in a xenograft mouse model.

Next, we investigated the interaction of PGRMC1 with enzymes involved in the cholesterol synthesis pathway such as CYP51, FDFT1, and SCD1. Further, the impact of PGRMC1 expression on lipid levels and expression of enzymes involved in lipid homeostasis was examined. Additionally, we assessed the role of PGRMC1 in key cancer-related signaling pathways including EGFR/HER2 and ERα signaling.

**Results:**

Overexpression of PGRMC1 resulted in significantly enhanced proliferation. PGRMC1 interacted with key enzymes of the cholesterol synthesis pathway, alters the expression of proteins, and results in increased lipid levels. PGRMC1 also influenced lipid raft formation leading to altered expression of growth receptors in membranes of breast cancer cells. Analysis of activation of proteins revealed facilitated ERα and EGFR activation and downstream signaling dependent on PGRMC1 overexpression in hormone receptor-positive breast cancer cells. Depletion of cholesterol and fatty acids induced by statins reversed this growth benefit.

**Conclusion:**

PGRMC1 may mediate proliferation and progression of breast cancer cells potentially by altering lipid metabolism and by activating key oncogenic signaling pathways, such as ERα expression and activation, as well as EGFR signaling. Our present study underlines the potential of PGRMC1 as a target for anti-cancer therapy.

## Background

With approximately 25% of all new cancer cases, breast cancer is the most common cancer in women [1] and responsible for the highest fraction of cancer death [2]. Therefore, the investigation of underlying mechanisms on molecular levels and the discovery of new therapy approaches are research goals of utmost significance.

Progesterone receptor membrane component 1 (PGRMC1) is a highly conserved protein, which is primarily found in the liver and kidney but also expressed in various tissues such as brain, breast, lung, pancreas, and reproductive tissues [3–5].

PGRMC1 has been confirmed to play a role in carcinogenesis especially in breast cancer and may therefore represent a target for cancer therapy [6]. In many studies, upregulation of PGRMC1 protein and mRNA was detected in malignancies including colon, lung, ovary, cervix, and breast [7–11]. Besides, PGRMC1 expression correlates with metastasis to lymph nodes, larger tumor size, and poorer overall- and tumor-free survival [9, 12]. Further, interactions of PGRMC1 or its homologous proteins with cytochrome P450 enzymes (CYPs) have been reported, for example by stably binding heme in its cytb5 related domain [3, 5, 13–15]. PGRMC1 leads to resistance against chemotherapeutic agents like doxorubicin, cisplatin, and paclitaxel [13, 16, 17]. Moreover, different authors discuss an involvement of PGRMC1 in cholesterol synthesis via interaction with CYPs [3, 5, 18]. The role of cholesterol in cancer is still not fully evaluated. Many studies describe an association of high plasma and endogenous cholesterol levels with (breast) cancer development and progression [19–21], pointing towards a major role in cancer. Elevated cholesterol and steroid levels may affect carcinogenesis in different ways, e.g., in saturating the increased requirement for membrane components due to abundant cell growth [22]. Furthermore, high cholesterol levels result in an increase in the size and number of lipid rafts. Since lipid rafts contain several signaling molecules, differences in lipid rafts are modulating signaling cascades [23, 24], such as EGFR and HER2 signaling and expression [25]. In addition, cholesterol is the precursor of steroid hormones like estradiol (E2), the important growth factor for hormone receptor-positive breast cancer [26].

The aim of the present study was to investigate the impact of PGRMC1 on lipid metabolism, lipid raft formation, and its contribution to breast cancer progression and cancer-associated signaling pathways in hormone receptor-positive (MCF7) and hormone receptor-negative (MDA-MB-231) cells. For this purpose, interaction of PGRMC1 with enzymes of the mevalonate pathway was evaluated. Subsequently, effects of PGRMC1 expression on cholesterol and lipid levels were investigated. A special focus was placed on PGRMC1-dependent expression and signaling of ERα and EGFR/HER2. To explore the impact of modified lipid and steroid metabolism (due to PGRMC1 expression), breast cancer cell growth was further explored by PGRMC1 overexpression and -silencing.

## Methods

### Cells and cell culture

MCF7, T47D, and MDA-MB-231 cells were purchased from the ATCC (Manassas, Virginia). Cells were maintained in RPMI 1640 medium (Thermo Fisher Scientific, Waltham, Massachusetts), supplemented with 10% (v/v) fetal bovine serum (Thermo Fisher Scientific, Waltham, Massachusetts), 100 units/mL penicillin/streptomycin (Thermo Fisher Scientific, Waltham, Massachusetts), and 0.025 mol/L HEPES (Thermo Fisher Scientific, Waltham, Massachusetts) in a humidified incubator at 37 °C with 5% CO_2_. Cells (passage number ≤ 25) were authenticated regularly by Microsynth AG (Balgach, Switzerland) using STRS analysis. The last authentication was performed on May 22, 2018.

### Transfection of cell lines

Cells were transfected with the expression vector pcDNA3.1/Hygro(+) (Thermo Fisher Scientific, Waltham, Massachusetts), containing 3x HA-tagged (3x human influenza hemagglutinin-tagged) PGRMC1, using Lipofectamine™ 2000 transfection reagent (Thermo Fisher Scientific, Waltham, Massachusetts) (MCF7/PGRMC1, T47D/PGRMC1 and MDA-MB-231/PGRMC1). As a control, we used cells transfected with the “empty” vector (MCF7/EVC, T47D/EVC, and MDA-MB-231/EVC). Stable transfection was verified by PCR, western blot, and immunofluorescence staining, to isolate PGRMC1-over-expressing clones.

### siRNA silencing of endogenous PGRMC1

For silencing of endogenous PGRMC1 in MCF7 cells, FlexiTube GeneSolution for PGRMC1 (Qiagen, Hilden, Germany) was used, containing four siRNAs that specifically target human PGRMC1 mRNA. Cells were harvested after cultivation for 24 h, 48 h, and 72 h at 37 °C to verify silencing by western blot analysis.

For MTT assays, cells were pre-incubated with siRNA against PGRMC1 for 24 h at 37 °C in cell culture flasks to silence the endogenous protein. Subsequently, the cells were seeded in 96-well plates and again treated with siRNA. Cell viability was measured after 24 h, 48 h, and 72 h at 37 °C of incubation.

### MTT assay

Cells (5 × 10^4^ cells per well) were seeded in triplicates in 96-well plates in complete medium. Cells were either grown (for different timespans) in full medium without or with treatment. Afterwards cells were incubated with 0.25 mg/ml MTT solution for 3 h. After 1 h of incubation with DMSO, absorption at 540 nm was determined with TECAN Spark®.

### Quantification of lathosterol and cholesterol

Cholesterol and lathosterol were quantified by gas chromatography-mass spectrometry analysis as described previously (Maier et al., 2009), with minor modifications.

### Western blot analysis

Samples for western blot analysis and the respective molecular weight marker were loaded onto Mini-PROTEAN® Precast Gel and separated via SDS-Page at 150 V. We activated the PVDF membrane with methanol. Transmission of proteins was performed for 16 h at 4 °C and 10 mA in blotting buffer. Afterwards, unspecific binding was blocked by incubation of the PVDF membrane with the transferred proteins with blocking solution for 1 h at room temperature. Primary antibody in respective concentration was added in blocking solution and incubated for 16 h at 4 °C. Subsequently, a secondary antibody was applied in 20% blocking solution at room temperature. Proteins were detected using Amersham™ ECL™ Western Blotting Detection Reagent.

### Co-immunoprecipitation

Immunoprecipitation of HA-tagged PGRMC1 and HA-tagged PGRMC1-variants was performed using the Pierce™ HA-Tag IP/Co-IP Kit according to the manufacturer’s instructions. Cells overexpressing GFP-tagged PGRMC1 were used as a negative control. Cell pellets were resuspended in Co-IP lysis buffer. An amount of 500-μg protein was incubated with anti-HA agarose slurry at 4 °C overnight. For elution, proteins were denatured in sample buffer at 95 °C for 5 min and the eluent was supplemented with 1 M DTT. The elution of PGRMC1 and mutual interaction partners was analyzed directly via mass spectrometry (explained in the supplements), SDS-PAGE, and western blot.

### Proximity ligation assay (PLA)

The PLA procedure was performed using the Duolink® PLA Kit. Cells were grown in chamber slides. Incubation with the primary antibody cocktail containing anti-PGRMC1 antibody and antibody against one of the possible interaction partners (or rabbit isotype IgG as negative control) was performed overnight at 4 °C.

Additionally, staining with anti-cytokeratin antibody for 1 h was performed after amplification. Afterwards, cells were stained with DAPI for 10 min and analyzed by fluorescence microscopy within 1 week.

### Reverse phase protein array (RPPA)

RPPA using Zeptosens technology was used for analysis of signaling protein expression and activity profiling.

RPPA assay images were analyzed using ZeptoVIEW Pro 3.1 array analysis software. Sample signals were quantified as protein-normalized, blank-corrected mean fluorescence intensities (NFI) of the single spots applying linear fits and interpolation to the mean of the four printed sample dilutions (eight spots per sample).

### qRT-PCR

RNA was isolated from a cell pellet of 0.5 **×** 10^6^ cells using the RNeasy Mini Kit (Qiagen, Hilden, Germany) according to the manufacturer’s specifications.

Reverse transcription of RNA into cDNA was performed with the Omniscript RT kit (Qiagen, Hilden, Germany) according to the manufacturer’s instructions. For qPCR, QuantiFast SYBR Green PCR kit (Qiagen, Hilden, Germany) and RT [2] qPCR Primer assays for ESR1, HER2, TFF1, Myc, CCND1, PGR, SCD, FASN, HMGS1, SREBF1, SREBF2, LDLR, ACAT1, and PDH (Qiagen, Hilden, Germany) were used according to the manufacturer’s specifications. qPCR was performed using the LightCycler® 480 System (Roche, Penzberg, Germany).

### Estradiol ELISA

Supernatants of MCF7/EVC and MCF7/PGRMC1 cells were analyzed for 17β-Estradiol (E2) concentrations using a commercially available kit (ab108667, Abcam plc, Cambridge, UK) according to the manufacturer’s specifications.

### Staining for lipid rafts and HER2

Co-staining of HER2 with lipid rafts was performed in PGRMC1 overexpressing MCF7 and MDA-MB-231 cells and their respective empty vector controls. Cells were seeded in a chamber slide for 24 h. Afterwards, the medium was removed, and the cells were incubated for another 24 h with medium containing stripped FCS and were then incubated for 24 h with medium containing normal FCS. Staining of lipid rafts was performed using Vybrant™ Alexa Fluor™ 488 Lipid Raft Labeling Kit. Afterwards, cells were fixed with 4% formaldehyde for 10 min. DAKO® protein block was used to block unspecific binding sites for 1 h. Following this, cells were stained with antibodies specific for HER2 (ab16901) over night at 4 °C followed by an anti-mouse secondary-antibody (Alexa Fluor 549 labeled) for 1 h. As negative control mouse isotype IgG was used. After this, staining with DAPI was performed. Subsequently cells were examined by fluorescence microscopy using Axioplan 2 Imaging (Carl Zeiss Microscopy GmbH, Jena, Germany). For analyzing the amounts of lipid rafts and HER2 via flow cytometry, cells were seeded in culture flasks and synchronized as described above. Staining and fixation was performed as described above. The emission (488 nm wavelength) was detected via high throughput flow cytometry (CyAn, Beckman Coulter, Brea, USA).

### Staining of lipid droplets

For visualizing of lipid droplets in PGRMC1 overexpressing MCF7, T47D, and MDA-MB-231 cells and their respective empty vector controls via fluorescence microscopy, the cells were grown in chamber slides for 24 h. Afterwards cells were stained with BODIPY™ 493/503 (Sigma-Aldrich, St. Louis, Missouri) solubilized in FCS-free medium and 2% BSA for 30 min. Cells were fixed with 4% formaldehyde for 10 min, stained with DAPI, and examined by fluorescence microscopy. For analyzing amounts of lipid droplets via flow cytometry, cells were grown for 24 h and harvested with trypsin. Staining was performed as described above. The emission (488-nm wavelength) was detected via high throughput flow cytometry (CyAn, Beckman Coulter, Brea, USA).

### Scatter plots of breast cancer microarray data

We obtained normalized microarray data (Affymetrix Human Genome U133A Array) from the Gene Expression Omnibus (GEO, NCBI) [27]. The samples were normalized using global scaling by the data set authors. We confirmed the value distribution using mean values and boxplots. Technical replicates were averaged. The values of a selected panel of reporters were correlated against a PGRMC1 reporter utilizing Spearman’s correlation.

### Xenograft models

NOD.CB17-Prkdc^scid^ (SCID) mice (female, 6-weeks old) were obtained from the Jackson Laboratory (Bar Harbor, Maine) and were bred in the SPF animal facility of the Institute of Genetics at the Biological Research Centre, Szeged, Hungary. Young adult SCID female mice were transplanted subcutaneously in the flank with 17β-estradiol pellet (containing biodegradable carrier-binder, 1.7 mg/pellet, 60-day release; SE-121, Innovative Research of America, Sarasota, Florida) under pentobarbital anesthesia. The next day, the mice were injected subcutaneously with 3 × 10^6^ tumor cells in the opposite flank. The mice were checked daily, and the tumor size was measured twice weekly. At the end of the experiment, the animals were euthanized, by pentobarbital overdose, and the tumors dissected.

### Treatment with simvastatin

For treatment with simvastatin, cells (10^5^ cells per well) were seeded in 96-well plates in complete medium for 24 h/37 °C. Afterwards, the medium was removed and the cells were incubated with 100, 50, 25, 12.5, 6.25, and 3.125 μg/mL simvastatin for MCF7 cells and 20, 10, 5, 2.5, 1.25, and 0.625 μg/mL simvastatin for MDA-MB-231. MTT assays were performed after 24 h, 48 h, and 72 h.

### Statistical analysis

All experiments were performed with several independent biological replicates and repeated a minimum of three iterations. Results are reported as means with standard deviation. The data were tested for normal distribution using Shapiro-Wilk and Kolmogorov-Smirnov test. Differences between groups were determined by unpaired Student’s *t* test. Statistical analysis was performed using R (RStudio) and IBM SPSS. Spearman’s *ρ* was calculated in R using normalized microarray data and was plotted as a scatterplot using the ggpubr R library. *p* < 0.05 was considered as statistically significant.

## Results

### PGRMC1 promotes viability of breast cancer cells and growth of xenograft tumors while PGRMC1 inhibition and downregulation reduce viability of breast cancer cells

**Fig. 1:**
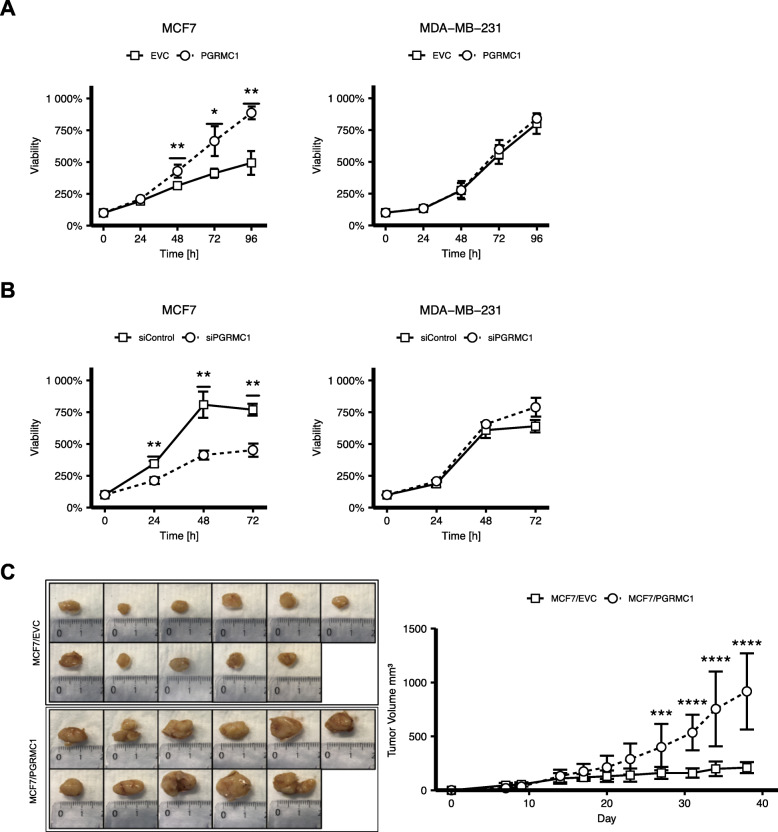
**a** Cell viability of MCF7/EVC and MCF7/PGRMC1 cells as well as MDA-MB-231/EVC and MDA-MB-231/PGRMC1 cells (*n* = 3). Viability was analyzed by MTT assay at *t* = 0 h, 24 h, 48 h, 72 h, and 96 h/37 °C. Values were normalized to *t* = 0 (100%). *p ≤ 0.05, **p ≤ 0.01 (Student’s *t* test, *n* = 3). **b** Cell viability of MCF7 and MDA-MB-231 cells, treated with siRNA against *PGRMC1* (siPGRMC1) and scrambled siRNA (siControl) (Student’s *t* test, *n* = 3). Viability was analyzed at *t* = 0 h, 24 h, 48 h, and 72 h/37 °C. Values were normalized to *t* = 0 (100%). *p ≤ 0.05, **p ≤ 0.01 (Student’s *t* test, *n* = 3). **c** Tumor volumes of immunodeficient mice bearing human breast cancer MCF7/EVC and MCF7/PGRMC1 xenografts. ***p ≤ 0.001, ****p ≤ 0.0001 (Student’s *t* test, *n* = 11 mice each group). Images of tumor tissue dissected from each mouse

As already shown in previous studies by us and others, PGRMC1 overexpression results in increased proliferation of tumor cells [28–30]. In accordance with these results, in our study, MCF7/PGRMC1 and T47D/PGRMC1 cells also profit from a significantly higher viability compared to the respective empty vector control cells (Fig. 1b, supplemental Figure 1A). For MDA-MB-231 cells overexpressing PGRMC1, no such effects can be observed (Fig. 1b). To further strengthen our theory, we examined the impact of PGRMC1 silencing on tumor proliferation by knocking down endogenous PGRMC1 expression. As hypothesized, the knockdown of PGRMC1 led to significantly decreased viability of MCF7 and T47D cells but not of MDA-MB-231 cells (Fig. 1a, supplemental Figure 1B).

To validate and strengthen the in vivo findings of Ruan et al. [30], to verify “our” cell models but also to extend the data to other ER-positive BC cells, we investigated effects of PGRMC1 overexpression on MCF7 and T47D breast cancer cell growth in a xenograft model. On that account, MCF7/PGRMC1 and T47D/PGRMC1 cells were injected into the flanks of immunodeficient mice. As control, we used EVC cells. Subsequently, the size of the developed tumor mass was measured. As assumed, mice injected with PGRMC1 overexpressing breast cancer cells matured significantly larger tumor masses, than mice injected with the respective EVC cells (Fig. 1c, supplemental Figure 1C).

### PGRMC1 interacts with proteins of the mevalonate pathway

As already shown in previous studies from different research groups, PGRMC1 might regulate cholesterol synthesis in different ways, e.g., by activating enzymes of the mevalonate pathway like CYP51/lanosterol demethylase or by binding to the proteins Insig and Scap, which span the endoplasmic reticulum and sense cholesterol levels [31, 32]. In our present study, we focused on this regulating influence and its possible involvement in PGRMC1-induced breast cancer promotion.

**Fig. 2:**
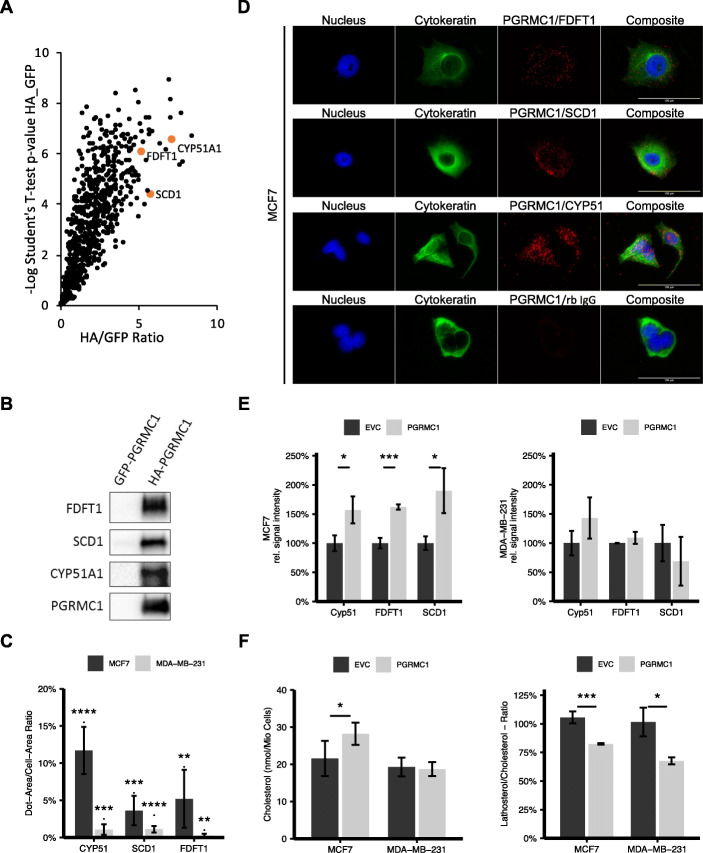
**a** Scatter plot of proteins with significantly higher intensities in PGRMC1-HA samples compared to PGRMC1-GFP samples identified by mass spectrometry. The most significant proteins exhibit very high value for Student’s *t* test difference HA_GFP and –log Student’s *t* test *p* value HA_GFP and are found in the upper right corner. Highlighted are proteins with important functions in steroid synthesis. **b** Detection of co-immunoprecipitated proteins CYP51A1, Stearoyl-CoA desaturase (SCD1), and FDFT1 by western blot. **c** Verification of the interactions via proximity ligation assay. Quantification of dots per cell. **d** Visualization via immunofluorescence microscopy. **e** Quantification of protein expression of CYP51, SCD1, and FDFT1 in MCF7/PGRMC1 cells and MDA-MB-231/PGRMC1 cells compared to their respective empty vector control by western blot. *p ≤ 0.05, ***p ≤ 0.001. **f** Detection of cholesterol and its precursor lathosterol in PGRMC1 overexpressing cells compared to the empty vector control cells with mass spectrometry *p ≤ 0.05, ***p ≤ 0.001 (Student’s *t* test, *n* = 3)

In order to get a broader view about the role of PGRMC1 in this context, we screened for potential PGRMC1 interaction partners by mass spectrometry analysis of proteins co-immunoprecipitated from whole cell lysates of MCF7 cells that had been transfected with PGRMC1-HA, utilizing an antibody directed against the HA-tag (Fig. 2a). Among proteins with higher significance, we found various potential interaction partners involved in the mevalonate pathway (e.g., SCD1, FDFT1, and CYP51A1) and cellular transport processes such as vesicle trafficking (e.g., Coatomer subunit beta and Coatomer subunit gamma-1) and nuclear export or import (e.g., Exportin-1, Exportin-2, Exportin-5, Exportin-7 or Importin-4 and Importin-5) processes. Since SCD1, FDFT1, and CYP51A1 indicate a high evidence for protein interaction with PGRMC1 and since they play an important role in cholesterol metabolism, we scrutinized these interactions. Interaction of PGRMC1 with SCD1, FDFT1, and CYP51A1 was confirmed by immunoprecipitating PGRMC1-HA in MCF7/PGRMC1 cells and by subsequently visualizing the respective interaction partners via western blot (Fig. 2b). To verify the observed interactions in different cell lines independently of PGRMC1 overexpression and immunoprecipitation, we performed proximity ligation assay of candidate proteins with endogenous PGRMC1 in MCF7 (Fig. 2c) and MDA-MB-231 cells (supplemental Figure 1B). Interactions between PGRMC1 and the respective enzymes are represented by single spots in fluorescence microscopy. While in MCF7 cells, a high number of spots per cell were visible for the interaction with CYP51, FDFT1, and SCD1, the low number of spots in MDA-MB-231 cells indicated no or little interaction (Fig. 2d). Interactions of PGRMC1 with FDFT1 and SCD1 were also observed in T47D cells (supplemental Figure 2B,C). Western blot analysis of protein expression of SCD1, FDFT1, and CYP51 revealed higher CYP51 and SCD1 protein levels in MCF7/PGRMC1 cells compared to MCF7/EVC, while no difference in MDA-MB-231/PGRMC1 cells could be observed compared to MDA-MB-231/EVC cells (Fig. 2e). These results implicate not only a direct interaction of PGRMC1 with SCD1, FDFT1, and CYP51, but also an increased PGRMC1-driven upregulation of these enzymes in estrogen receptor-positive cells, that appeared absent in hormone receptor-negative cells.

### Overexpression of PGRMC1 leads to higher levels of cholesterol in hormone receptor-positive breast cancer cells

We hypothesized that the interaction of PGRMC1 with enzymes of the mevalonate pathway might alter their function and thus affects cholesterol synthesis, resulting in elevated cholesterol levels, which may provide energy and components supporting cancer metabolism. Therefore, we measured intracellular cholesterol levels in synchronized PGRMC1 overexpressing and empty vector control MCF7 and MDA-MB-231 cells via mass spectrometry (Fig. 2f). Overexpression of PGRMC1 in MCF7 cells caused a significant increase (*p* < 0.05) of intracellular cholesterol levels compared to the empty vector control, while no difference in MDA-MB-231/PGRMC1 cells was observed (Fig. 2f). Additionally, levels of lathosterol, a precursor of cholesterol, were measured (Fig. 2f). For MCF7/PGRMC1 cells, we detected a significantly decreased ratio compared to MCF7/EVC cells. Interestingly, a significantly decreased ratio of lathosterol/cholesterol in MDA-MB-231/PGRMC1 cells was observed compared to MDA-MB-231/EVC cells, pointing towards a small influence of PGRMC1 on cholesterol de novo synthesis in these cells. The data reveal an impact of PGRMC1 on de novo synthesis of cholesterol regarding cholesterol levels and enzymatic turnover.

### Upregulation of ERα, ERα downstream targets, and E2 levels mediated by PGRMC1

**Fig. 3:**
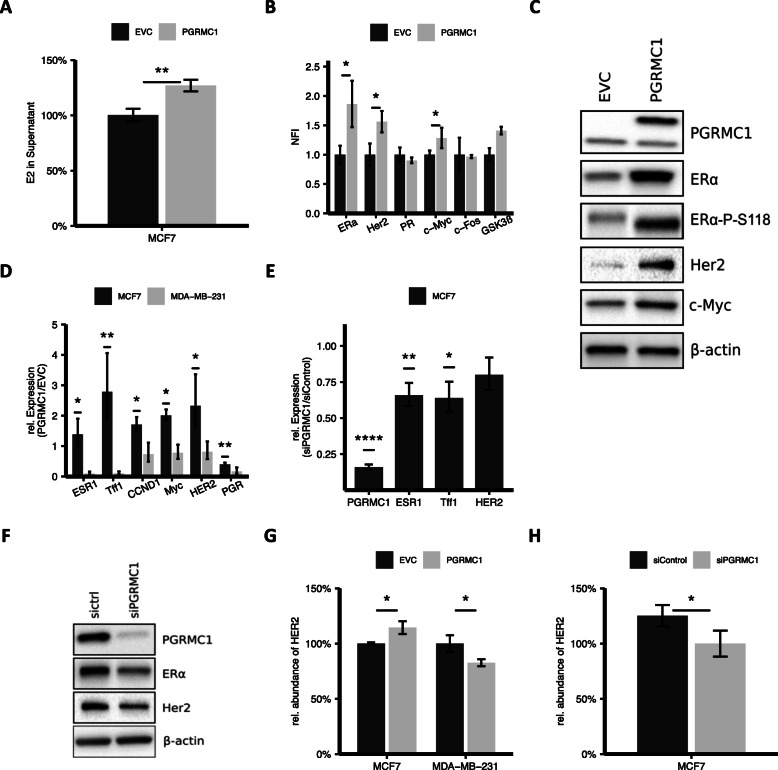
**a** Amount of E2 in the supernatant of MCF7/PGRMC1 cells compared to the empty vector control after 48 h, detected with ELISA. **p ≤ 0.01. **b** NFI (blank-corrected mean fluorescence intensity) ratio of protein expression of ERα, Her2, PR, c-Myc, and c-Fos analyzed by RPPA. Protein expression was normalized to MCF7/EVC and protein expression measured in MCF7/EVC cells was set to 1. Up-/downregulation of protein expression in MCF7/PGRMC1 cells were calculated. *p ≤ 0.05 (Student’s *t* test, *n* = 3). **c** Western blot analysis of ERα, Her2, and c-Myc protein levels in MCF7/EVC and MCF7/PGRMC1 cells. Representative picture of 3 independent analyses. **d** qRT-PCR analysis of *ESR1*, *TFF1*, *HER2*, *CCND1*, *Myc*, and *PGR* mRNA expression in MCF7/EVC and MCF7/PGRMC1 cells, MDA-MB-231/EVC and MDA-MB-231/PGRMC1 cells. *p ≤ 0.05, ***p ≤ 0.001 (Student’s *t* test, *n* = 3). **e** qRT-PCR analysis of *PGRMC1*, *ESR1*, *HER2*, and *TFF1* mRNA expression in MCF7 siCtrl and MCF7 siPGRMC1 cells. *p ≤ 0.05, **p < 0.01, ****p ≤ 0.0001 (Student’s *t* test, *n* = 3). **f** Western blot analysis of ERα and Her2 protein levels in MCF7 siCtrl and MCF7 siPGRMC1 cells. Representative blot from 3 independent analyses. **g** Quantification of HER2 protein in membranes of unpermeabilized MCF7/EVC and MCF7/ PGRMC1 cells, MDA-MB-231/EVC and MDA-MB-231/PGRMC1 cells, and MCF7 siCtrl and MCF7 siPGRMC1 cells **(h)** via flow cytometry. *p ≤ 0.05 (Student’s *t* test, *n* = 3)

Since cholesterol is the precursor for steroid hormones, we assumed that enhanced cholesterol synthesis may affect E2 levels. E2 plays an essential role in hormone receptor-positive breast cancer, e.g., by activating ERα which is leading to tumor proliferation. E2 levels were determined in the supernatant of MCF7/PGRMC1 cells by ELISA (Fig. 3a). Consistent with the higher amounts of cholesterol in MCF7/PGRMC1 cells, we found significantly increased levels of E2 in the supernatant of MCF7/PGRMC1 cells in comparison to MCF7/EVC cells. To analyze the effect of higher E2 levels in MCF7/PGRMC1 cells on breast cancer signaling, we determined the expression of different proteins known to play a role in key signaling cascades in breast cancer via reverse phase protein array technology (RPPA) (Fig. 3b). RPPA analysis revealed significantly (*p* < 0.05) elevated expression of ERα in MCF7/PGRMC1 cells compared to MCF7/EVC cells (Fig. 3b). Subsequently higher levels of HER2 and c-Myc proteins, whose expression depend on the transcriptional activity of ERα, were observed while c-Fos and PR levels were not altered (Fig. 3b). To verify the results from RPPA, western blots were performed to detect protein expression of ERα, HER2, and c-Myc (Fig. 3c). In MCF7/PGRMC1 cells, expression of ERα, HER2, and c-Myc is increased. Because E2 activates ERα and our previous studies have demonstrated higher E2 levels in MCF7/PGRMC1 cells compared to MCF7/EVC (Fig. 3a), we analyzed ERα phosphorylation at S118 (ERα-P-S118), which was also significantly increased (*p* < 0.01) in MCF7/PGRMC1 cells compared to MCF7/EVC (Fig. 3c). Additionally, we performed qPCR analysis of mRNA expression for ESR1, Tff1, HER2, CCND1, Myc, and PGR in the PGRMC1 overexpressing cell lines in comparison to the empty vector control (Fig. 3d, supplemental Figure 3B). In MCF7/PGRMC1 and T47D/PGRMC1 we detected higher mRNA levels for ESR1 and the ERα-dependent gene trefoil factor 1 (Tff1), CCND1 and Myc as reporter genes for ERα activation compared to MCF7/EVC and T47D/EVC. Interestingly, mRNA levels of PGR were significantly lower in the PGRMC1 overexpressing cells compared to their empty vector control. To further consolidate our hypothesis, we significantly silenced (*p* < 0.01) PGRMC1 expression by siPGRMC1 (Fig. 3e). As expected, the expression of ERα, ESR1, and Tff1 were significantly downregulated (Fig. 3e), albeit no significant upregulation was detected for mRNA levels of HER2 pointing towards a post-transcriptional regulation of HER2 levels by PGRMC1 (Fig. 3e). In accordance, western blot analysis revealed decreased expression of ERα and HER2 in MCF7/siPGRMC1 (Fig. 3f). Previous studies revealed that HER2 overexpression causes deformation of the cell membrane and a subsequent disruption of epithelial features independent of receptor signaling [25, 33]. We demonstrated higher HER2 expression on the surface of non-permeabilized MCF7/PGRMC1 cells compared to MCF7/EVC cells using flow cytometry (Fig. 3g). Similarly, HER2 levels were reduced on the surface of MCF7/siPGRMC1 cells (Fig. 3h). MDA-MB-231/PGRMC1 cells even showed lower expression of HER2 compared to MDA-MB-231/EVC cells (Fig. 3g).

### PGRMC1 overexpressing breast cancer cells show higher amounts of neutral lipids and lipid droplets

Lipid droplets recently emerged as new organelles not only due to their role in energy storage, but also as modulators of cell signaling and lipid homeostasis in several diseases including breast cancer [34–36].

**Fig. 4:**
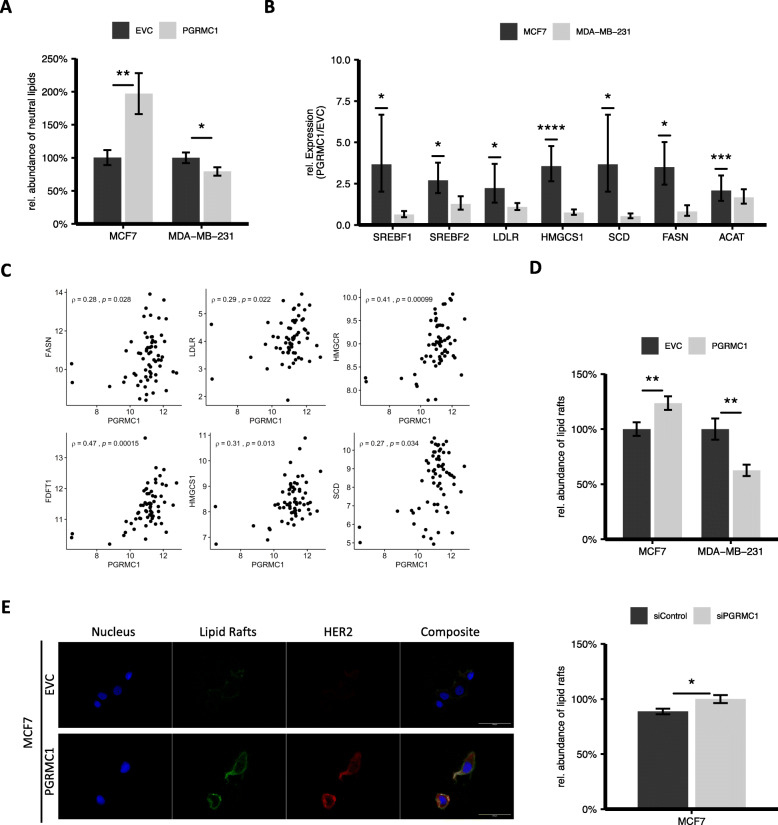
**a** Detection of neutral lipids and lipid droplets in MCF7/EVC and MCF7/PGRMC1, MDA-MB-231/EVC and MDA-MB-231/PGRMC1 cells by BODIPY® staining and quantification via flow cytometry. *p ≤ 0.05, **p ≤ 0.01. (Student’s *t* test, *n* = 3). **b** qRT-PCR analysis of SREBF1, SREBF2, LDLR, HMGS1, SCD, FASN, ACAT mRNA expression in MCF7/PGRMC1 and MDA-MB-231/PGRMC1 cells compared to the respective EVC cells. *p ≤ 0.05, **p ≤ 0.01, ***p ≤ 0.001 (Student’s *t* test, *n* = 3). **c** Spearman’s correlation between the PGMRC1 expression level and various expression levels of proteins (FASN, FDFT1, HMGCS1, HMGCR, LDLR, SCD) involved in lipid metabolism. Data obtained from normalized microarray data (Affymetrix Human Genome U133A Array) of 63 hormone receptor-positive breast cancer tissue samples. **d** Detection of lipid rafts in cell membranes of MCF7/EVC and MCF7/PGRMC1 cells, MDA-MB-231/EVC and MDA-MB-231/PGRMC1 cells, and MCF7 siCtrl and MCF7 siPGRMC1 cells by Vybrant™ Alexa Fluor™ 488 and subsequent quantification via flow cytometry. *p ≤ 0.05, **p ≤ 0.01 (Student’s *t* test, *n* = 3). **e** Immunofluorescence staining with Vybrant™ Alexa Fluor™ 488, fluorescence immunocytochemistry for HER2, and nuclear staining with DAPI. 63-fold magnification. Cells were grown on chamber slides for 24 h

By altering cholesterol levels in breast cancer cells, PGRMC1 could have a major influence on tumor growth via an enhanced lipid droplet formation in hormone receptor-positive breast cancer. To quantify the amount of neutral lipids, PGRMC1 overexpressing cell lines and their respective empty vector control were examined by BODIPY® staining of neutral lipids respectively lipid droplets. Subsequent flow cytometry analysis showed that PGRMC1 overexpressing hormone receptor-positive cells have a significantly higher amount of neutral lipids in comparison to the empty vector control (Fig. 4a, supplemental Figure 4A). Interestingly, we found significantly lower levels of lipids in MDA-MB-231/PGRMC1 cells compared to MDA-MB-231/EVC (Fig. 4a). Our results point towards an upregulation of lipid synthesis due to PGRMC1 overexpression in hormone receptor-positive breast cancer, which might lead to enhanced tumor growth.

### PGRMC1 fuels endogenous lipid synthesis and lipid uptake and upregulates enzymes of the cholesterol metabolism

Besides the direct interaction of PGRMC1 with enzymes of the mevalonate pathway, the influence of PGRMC1 on lipid metabolism might be explained by increased mRNA expression of enzymes involved in endogenous and exogenous lipid metabolism.

Quantitative PCR analysis revealed increased levels of mRNA for SREBF1, SREBF2, LDLR, HMGS1, SCD, FASN, and ACAT1 in MCF7/PGRMC1 cells compared to MCF7/EVC cells (Fig. 4b, supplemental Figure 4B). These enzymes are not only key players in cholesterol and fatty acid synthesis, but also upregulated in breast cancer and they are associated with a worse outcome. In MDA-MB-231 cells, PGRMC1 overexpression did not result in higher expression of the abovementioned proteins (Fig. 4b). To show the increasing effect of PGRMC1 on expression of enzymes of the lipid metabolism, we obtained normalized microarray data of 63 hormone receptor-positive breast cancers tissue samples [37]. Spearman’s correlation between the PGMRC1 expression level and various expression levels of proteins (FASN, FDFT1, HMGCS1, HMGCR, LDLR, SCD) indicated positive correlations between PGRMC1 and the respective enzymes in luminal A breast cancer tissue samples (Fig. 4c). Our findings advert to a complex and diverse impact of PGRMC1 on lipid homeostasis in breast cancer.

### PGRMC1 enhances expression of lipid rafts in cell membranes of breast cancer cells

Lipid rafts are cholesterol-rich microdomains in cell membranes, which have functions in cell proliferation and growth, membrane trafficking, metastasis, and apoptosis [23, 24, 38]. Furthermore, lipid raft formation in cell membranes is influenced by FDFT1 activity [39]. Since lipid rafts play a role in breast cancer progression and due to the fact that (a) PGRMC1 overexpressing hormone receptor-positive breast cancer cells have higher amounts of cholesterol and that (b) PGRMC1 interacts with FDFT1, we determined the abundance of lipid rafts in MCF7 and MDA-MB-231 cells with PGRMC1 overexpression and respective empty vector control as well as in MCF7 cells treated with siRNAs directed against PGRMC1, to knockdown PGRMC1 (Fig. 4d). Cells were stained with Vybrant™ Alexa Fluor™ 488 Lipid Raft Labeling Kit and detected by flow cytometry. MCF7/PGRMC1 cells showed significantly higher levels of lipid rafts compared to the respective empty vector control (Fig. 4d, upper). In addition, we found significantly lower expression of lipid rafts when endogenous PGRMC1 was knocked down in MCF7 cells (Fig. 4d, lower). Interestingly, lipid rafts were decreased in PGRMC1 overexpressing MDA-MB-231 cells (Fig. 4d).

Elevated proliferation mediated by lipid rafts is, among others, attributed to modulation of signaling functions of growth factor receptors like the ErbB (HER) receptor family.

Since we found higher expression of HER2 in the membrane of PGRMC1 overexpressing MCF7 cells (Fig. 3g), we analyzed the HER2 expression in lipid rafts in more detail.

PGRMC1 overexpressing MCF7 and MDA-MB-231 cells and respective empty vector control cells were co-stained for HER2 and lipid rafts (Fig. 4e, supplemental Figure 3C). Especially in MCF7/PGRMC1 cells, we found a strong co-localization of HER2 in lipid rafts (Fig. 4e).

### PGRMC1 influences activation of EGFR signaling

**Fig. 5:**
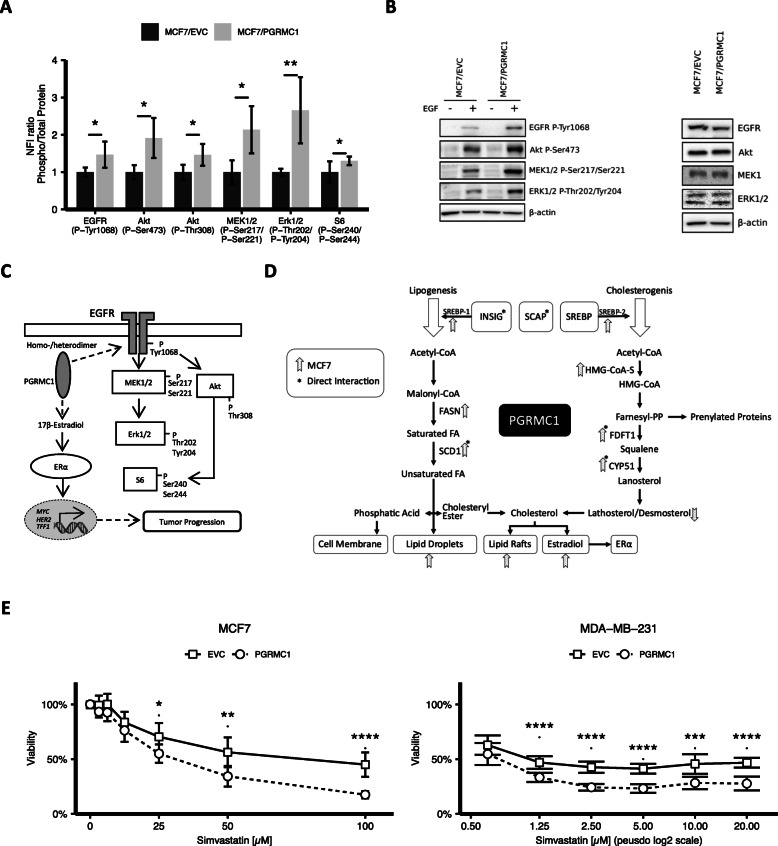
**a** Protein phosphorylation of EGFR P-Tyr1068, Akt P-Ser473, Akt P-Thr308, MEK1/2 P-Ser217/Ser221, Erk1/2 P-Thr202/Tyr204, and S6 P-Ser240/Ser244 analyzed by RPPA. NFI (blank-corrected mean fluorescence intensity) ratio of phospho-protein/total protein was calculated, normalized to MCF7/EVC, and ratio in MCF7/EVC cells was set to 1. Up-/downregulation of protein phosphorylation in MCF7/PGRMC1 cells was calculated. *p ≤ 0.05, **p ≤ 0.01 (Student’s *t* test, *n* = 3). **b** Protein phosphorylation of EGFR P-Tyr1068, Akt P-Ser473, MEK1/2 P-Ser217/Ser221, and Erk1/2 P-Thr202/Tyr204 verified by western blot analysis. Cells were treated with EGF (10 ng/mL) for 10 min/37 °C. Representative blot of 3 independent analyses**.** Total protein expression of EGFR, Akt, MEK1/2, and Erk1/2 verified by western blot analysis. Representative blot of 3 independent analyses shown. **c** PGRMC1 mediates phosphorylation of EGFR and its downstream targets and upregulates E2 levels, ERα expression, and ERα-target genes. EGFR phosphorylation activates the MAPK signaling cascade (including MEK1/2-, ERK1/2-, and S6-phosphorylation) and PI3K signaling cascade (including Akt- and S6-phosphorylation). Phosphorylation of S6 induces transcription of genes, involved in the regulation of cell cycle progression, cell proliferation, and glucose homeostasis. ERα translocates into the nucleus upon ligand-dependent or ligand-independent activation and acts as a transcription factor to transcribe genes involved in tumor progression. **d** Overview of the influence of PGRMC1 in cholesterol and lipid metabolism. **e** MCF7/EVC and MCF7/PGRMC1 cells were treated with 100 μM, 50 μM, 25 μM, 12.5 μM, 6.25 μM, and 3.175 μM simvastatin and respective DMSO control. MDA-MB-231/EVC and MDA-MB-231/PGRMC1 cells were treated with 20 μM, 10 μM, 5 μM, 2.5 μM, 1.25 μm, and 0.625 μM simvastatin and respective DMSO control. Viability was analyzed by MTT assay at *t* = 24 h, *t* = 48 h, *t* = 72 h and 37 °C. Depicted are results after 48 h of treatment. Viability is normalized on the DMSO control. p values were adjusted using the Bonferroni correction (*n*_doses_ = 6; *n*_replicates_ = 9)

Another important member of the ErbB receptor family, which plays a major role in breast cancer signaling, is the EGFR. Several studies suggest that PGRMC1 may promote EGFR phosphorylation and activation [8, 9, 13, 40]. The hypothesis of PGRMC1 enhancing EGFR signaling was investigated by reverse phase protein array (RPPA) with a focus on phosphorylation of EGFR and its downstream targets in MCF7/PGRMC1 and MCF7/EVC cells (Fig. 5a). Our results point towards an increased phosphorylation of EGFR (p-Tyr1068), Akt (p-Ser473 and p-Thr308), MEK1/2 (p-Ser217/Ser221), ERK1/2 (p-Thr202/Tyr204), and S6 (p-Ser240/Ser244) in PGRMC1/MCF7 cells compared to EVC cells (Fig. 5a). In combination with our results from immunofluorescence staining, this suggests that there might exist a powerful link between PGRMC1 expression and activation of oncogenic signaling pathways in MCF7 cells (Fig. 5c).

To verify the RPPA results, we performed western blot analysis of EGFR signaling induced with EGF (Fig. 5b). Phosphorylation of EGFR, Akt, MEK1/2, and ERK1/2 was observed (Fig. 5b). Compatible, significantly elevated levels of EGFR (p-Tyr1068), Akt (p-Ser473), MEK1/2 (p-Ser217/Ser221), and ERK1/2 (p-Thr202/Tyr204) were monitored in MCF7/PGRMC1 cells. In contrast, expression levels of total protein did not vary significantly (Fig. 5c). MDA-MB-231 showed no difference in expression levels of EGFR (p-Tyr1068), Akt (p-Ser473), MEK1/2 (p-Ser217/Ser221), and ERK1/2 (p-Thr202/Tyr204), suggesting a subordinated role of PGRMC1 in EGFR signaling in triple-negative breast cancer (supplemental Figure 4A, 4B).

### Cholesterol and fatty acid depletion induced by statins reverses the growth benefit interceded by PGRMC1

Our findings suggest a complex and broad role of PGRMC1 in cholesterol and lipid metabolism (Fig. 5d). Based on our research concerning the influence of PGRMC1 on lipid homeostasis and increased viability of PGRMC1 overexpressing cells, we hypothesized that a higher lipid synthesis might lead to a survival benefit of PGRMC1 overexpressing cells.

To verify this hypothesis, we treated PGRMC1 overexpressing MCF7 and MDA-MB-231 cells and the respective controls with different concentrations of simvastatin, a competitive inhibitor of HMG-CoA reductase, and performed subsequent viability assays (Fig. 5d).

Interestingly, contrary to expectations, inhibition of HMG-CoA reductase and following depletion of cholesterol not only assimilated viability in MCF7/PGRMC1 cells compared to MCF7/EVC cells, but even led to inferior viability. This suggests a higher dependence of PGRMC1 overexpressing cells on cholesterol. Intriguingly, MDA-MB-231 cells with PGRMC1 overexpression reacted similar to MCF7 cells (Fig. 5d).

## Discussion

Although previous studies report on the proliferative effect of PGRMC1 in breast cancer, little is known about the mechanisms by which PGRMC1 effects carcinogenesis. Therefore, our present study focuses on the modifying function of PGRMC1 on lipid metabolism and oncogenic signaling. Evidence is pointing towards a meaningful impact of modified lipid metabolism in breast cancer progression and metastasis [41–44]. Although one of the most relevant mechanisms of energy usage of cancer cells is their increase in glucose uptake and their use of non-oxidative glycolysis, also known as Warburg effect, breast cancer cells upregulate lipid de novo synthesis and the uptake of free fatty acids and low-density lipoproteins [44, 45]. Our findings suggest the function of PGRMC1 as an important enhancer especially of lipid synthesis resulting in oncogenic signaling and tumor progression. For the first time, we detected enhanced mRNA expression of proteins regulating lipid synthesis and uptake in PGRMC1 overexpressing hormone receptor-positive MCF7 and T47D cells resulting in significantly higher lipid levels in MCF7/PGRMC1 and T47D/PGRMC1 cells compared to the empty vector control cells. Further, we could demonstrate that PGRMC1 interacts with CYP51, FDFT1, and SCD1, which are major players in lipogenesis. Interestingly, these interactions are less pronounced in MDA-MB-231 cells. An explanation for the lower interaction might be that triple-negative breast cancer cells have been reported to cover their needs for lipids via the uptake of exogenous fatty acids in contrast to performing lipid de novo synthesis [44, 46].

A possible result of the detected interactions between PGRMC1 and CYP51, FDFT1, and SCD1 could be the increase of cholesterol and neutral lipid levels in MCF7/PGRMC1 and T47D/PGRMC1 cells. Since cholesterol is the precursor of steroid hormones like estradiol, elevated levels of cholesterol may subsequently lead to higher levels of estradiol as indicated by our measurements in the supernatant of MCF7/PGRMC1 cells of this scenario. One consequence could be that PGRMC1 promotes tumor progression by upregulation of ERα protein and ESR1 mRNA directly via a transcriptional mechanism or indirectly via elevated steroid synthesis. Since various studies showed an upregulation of steady-state ERα levels by long-term exposure to E2 [47], increased levels of ERα plus a simultaneous autocrine/paracrine activation by E2 may trigger a proliferative cycle support in tumor growth. For the first time, we also observed that PGRMC1 impacts on lipid rafts, another regulator of cancer progression. Lipid rafts are important, e.g., in modulation of membrane geometry, lateral movement of molecules, and signal transduction [23, 48]. We observed increased lipid raft formation in PGRMC1 overexpressing hormone receptor-positive breast cancer cells. The co-localization of HER2 in lipid rafts, also reported by other research groups [49, 50], may influence EGFR signaling. Zhuang et al. reported an EGF-induced and constitutive signaling via the Akt serine-threonine kinase and subsequent survival in cancer cells [51]. Furthermore, EGFR and HER2 localization in lipid rafts is discussed to play a role in cancer cell drug resistance, e.g., regarding treatment with trastuzumab or tyrosine kinase inhibitors [49, 50]. On the other hand, Orr et al. showed that altered cholesterol levels modify the mobility of EGFR in the cell membrane leading to its decreased activation due to reduced dimerization of EGFR monomeres [25]. The relevant role of PGRMC1 in promoting phosphorylation and activation of receptors for example by heme-dependent PGRMC1 dimerization has already been reported [8, 9, 13, 40]. Here, elevated phosphorylation levels of EGFR and its downstream targets in MCF7/PGRMC1 cells were discovered. The crosstalk between EGFR/Her2 and ERα signaling cascades has often been reported, whereby ERα can induce the E2-dependent activation of the EGFR signaling pathway by promoting phosphorylation of Akt (P-Ser473) via the non-genomic pathway. Alternatively, ERα activation can be accomplished independently of estrogens by EGFR-activated MAPK-signaling or PI3K pathway [52, 53]. In the current study, we demonstrate that both MAPK and PI3K pathway components (i.e., MEK1/2, ERK1/2, and AKT) are activated in PGRMC1 overexpressing MCF7 cells. This may lead to increased ERα activation and finally to increased cancer proliferation. Additionally, ERα and HER2 correlate positively in HER2 non-overexpressing breast cancer [54, 55]. Hence, higher levels of ER in MCF7/PGRMC1 cells could lead to higher expression of HER2. However, the influence of PGRMC1 on EGFR/HER2 signaling in lipid rafts and its impact on tumor progression requires further studies.

Due to the role of the mevalonate pathway and its dual role in cholesterol synthesis and prenylation of signaling proteins, statins have been tested as anti-cancer drugs. Statins block the HMG-CoA reductase, the gatekeeper of the mevalonate pathway. We speculated due to increased activation of the mevalonate pathway and due to higher cholesterol and neutral lipids production that PGRMC1 overexpressing cells may be more dependent on the mevalonate pathway. Hence, they might be more susceptible to statin treatment [56–60]. For the first time, we detected that MCF7/PGRMC1 and MDA-MB-231/PGRMC1 cells are more sensitive to treatment with simvastatin compared to the respective controls. We assume that PGRMC1 overexpression leads to higher dependence on cholesterol and fatty acids of cancer cells due to an alteration of fatty acid metabolism, by enhanced driving of the mevalonate pathway and related synthesis of the isoprenoids geranylgeranyl pyrophosphate (GGPP) and farnesyl pyrophosphate (FPP) [61, 62], e.g., leading to inhibition of small Rho GTPase prenylation [63].

Indeed, PGRMC1 might also reduce viability of breast cancer cells under treatment with statins, because PGRMC1 is known to interact with CYP enzymes [3, 5, 13–15]. Specifically, inhibition of cytochromes P450 could increase the concentration of simvastatin, since statins are metabolized by CYP3A4.

Hence, PGRMC1 overexpressing tumors may be an interesting target for additional cholesterol lowering therapy.

## Conclusion

We demonstrate that PGRMC1 mediates progression of breast cancer cells potentially by altering cholesterol and lipid metabolism and activating key drivers of tumor progression in breast cancer, namely ERα expression and activation, as well as EGFR signaling. Our data underline the contribution of PGRMC1 to especially hormone receptor-positive breast cancer pathogenesis in vitro and in vivo and suggest its potential as a target for anti-cancer therapy.

## Supplementary information

**Additional file 1.**

## Data Availability

The microarray data analyzed during the current study are available in the Gene Expression Omnibus under the ID GSE129560. [https://www.ncbi.nlm.nih.gov/geo/query/acc.cgi?acc=GSE129560]. The remaining datasets used and analyzed during the current study are available from the corresponding author on reasonable request.

## Abbreviations

ACAT1: Acetyl-Coenzyme A acetyltransferase 1
Akt: Protein kinase B
BODIPY: Difluoro {2-[1-(3,5-dimethyl-2H-pyrrol-2-ylidene-N)ethyl]-3,5-dimethyl-1H-pyrrolatoN} boron
BSA: Bovine serum albumin
CK-2: Casein kinase-2
CYP: Cytochromes P450
DAPI: 4,6-Diamidin-2-phenylindol
E2: 17β-Estradiol
EGF: Epidermal growth factor
EGFR: Epidermal growth factor receptor
ELISA: Enzyme-linked immunosorbent assay
Erk1/2 (MAPK p44/42): extracellular signal-regulated kinases
ERα: Estrogen receptor α
FASN: Fatty acid synthase
FCS: Fetal calf serum
GFP: Green fluorescent protein
HA: Hemagglutinin
Her2: Human epidermal growth factor receptor-2
HMGS: 3-Hydroxy-3-methylglutaryl coenzyme A synthase
INSIG: Insulin-induced gene protein
LDLR: Low-density lipoprotein receptor
MEK1 (MAP2K1): Mitogen-activated protein kinase kinase 1
MEK2 (MAP2K2): Mitogen-activated protein kinase kinase 2
NFI: Blank-corrected mean fluorescence intensity
PGRMC1: Progesterone receptor membrane component-1
PR: Progesterone receptor
RPPA: Reverse phase protein array
SCAP: SREBP cleavage-activating protein
SCD1: Stearoyl-CoA desaturase1
FDFT1: Farnesyl-diphosphate farnesyltransferase 1 (Squalene Synthase)
SREBP1/2: Sterol regulatory element-1/2
TFF1: Trefoil factor 1
