# Correction to: Progesterone receptor membrane component 1 regulates lipid homeostasis and drives oncogenic signaling resulting in breast cancer progression

**DOI:** 10.1186/s13058-020-01383-7

**Published:** 2021-01-28

**Authors:** Hannah Asperger, Nadia Stamm, Berthold Gierke, Michael Pawlak, Ute Hofmann, Ulrich M. Zanger, Annamaria Marton, Robert L. Katona, Andrea Buhala, Csaba Vizler, Jan-Philipp Cieslik, Zaklina Kovacevic, Des R. Richardson, Eugen Ruckhäberle, Dieter Niederacher, Tanja Fehm, Hans Neubauer, Marina Ludescher

**Affiliations:** 1 Department of Obstetrics and Gynecology, University Hospital and Medical Faculty of the Heinrich-Heine University Duesseldorf, Life Science Center, Merowingerplatz 1A, 40225 Düsseldorf, Germany; 2 NMI TT Pharmaservices, Protein Profiling, Reutlingen, Germany; 3 Dr. Margarete Fischer-Bosch Institute of Clinical Pharmacology and University of Tübingen, Stuttgart, Germany; 4 Institute of Biochemistry, Biological Research Centre, Szeged, Hungary; 5 Institute of Genetics, Biological Research Centre, Szeged, Hungary; 6 School of Medical Sciences, Faculty of Medicine and Health, Medical Foundation Building, University of Sydney, Sydney, New South Wales 2006 Australia; 7 Centre for Cancer Cell Biology and Drug Discovery, Griffith Institute for Drug Discovery, Griffith University, Brisbane, Queensland 4111 Australia

**Correction to: Breast Cancer Res (2020) 22:75**

**https://doi.org/10.1186/s13058-020-01312-8**

After publication of the original article [1], the author reported that the 12th and 13th authors, namely Zaklina Kovacevic and Des R. Richardson, were accidently omitted. Both these authors have now been added to the author group and are now presented correctly.

## References

[1] Asperger H (2020). Progesterone receptor membrane component 1 regulates lipid homeostasis and drives oncogenic signaling resulting in breast cancer progression. Breast Cancer Res.

