# Supplementary material for: Progesterone receptor membrane component 1 regulates lipid homeostasis and drives oncogenic signaling resulting in breast cancer progression
Source: Breast Cancer Res. 2020 Jul 13;22:75. doi: 10.1186/s13058-020-01312-8 (PMC7359014; doi:10.1186/s13058-020-01312-8)
Supplement: Supplementary file 1 — Additional file 1. [file 13058_2020_1312_MOESM1_ESM.pdf]

Supplements

Methods

Quantification of lathosterol and cholesterol

Cholesterol and lathosterol were quantified by GC-MS analysis as described previously (Maier et al., 2009), with minor modifications.

Briefly, the cell pellet was extracted with hexane:2-propanol 3:2 v/v, spiked with internal standards [$^2H_5$]cholesterol and [$^2H_7$]lathosterol and evaporated to dryness under nitrogen. The residue was saponified with 1 M NaOH in 90% ethanol for 1 h at 70 °C and subsequently extracted with cyclohexane. The organic phase was evaporated and the *tert*-butyldimethylsilyl derivatives prepared by addition of 20 µl of N-*tert*-butyldimethylsilyl-N-methyltrifluoroacetamide (MBDSTFA) and 20 µl of DMF. Cholesterol and lathosterol were measured by GC-MS on a 5975C inert XL MSD, coupled to a 7890A GC (Agilent) in selected ion monitoring (SIM) mode at m/z 443.4, [$^2H_5$]cholesterol at m/z 448.4, and [$^2H_7$]lathosterol at m/z 450.4. Calibration samples prepared directly from the working solutions, were worked up as described above, and analyzed together with the unknown samples. Calibration curves based on internal standard calibration were obtained by weighted (1/x) linear regression for the peak-area ratio of the analyte to the respective internal standard against the amount of the analyte. The concentration of the analytes in unknown samples was obtained from the regression line.

PGRMC1 was immunoprecipitated from four individual replicates of MCF7/PGRMC1-HA cells. As a negative control, GFP-labelled MCF7/PGRMC1 cells (MCF7/PGRMC1-GFP) were used. Resulting protein preparations were shortly separated in a 4-12% polyacrylamide gel (about 4 mm running distance), silver stained and processed as

Mass spectrometry of PGRMC1 interaction partners

PGRMC1 was immunoprecipitated from four individual replicates of MCF7/PGRMC1-HA cells. As a negative control, GFP-labelled MCF7/PGRMC1 cells (MCF7/PGRMC1-GFP) were used. Resulting protein preparations were shortly separated in a 4-12% polyacrylamide gel (about 4 mm running distance), silver stained and processed as
previously described. Briefly, samples were destained, reduced with dithiothreitol, alkylated with iodoacetamide, digested with trypsin peptides extracted from the gel and finally resuspended in 0.1 % trifluoroacetic acid. Subsequently, the samples were analyzed on a liquid chromatography coupled electrospray ionization Orbitrap mass spectrometer. An Ultimate 3000 Rapid Separation Liquid Chromatography System was used for peptide separation: peptides were initially pre-concentrated on a trap column (Acclaim PepMap100, 3 µm C18 particle size, 100 Å pore size, 75 µm inner diameter, 2 cm length) at a flow rate of 6 µl/min for ten minutes using 0.1 % TFA as mobile phase and thereafter separated on an analytical column (Acclaim PepMapRSLC, 2 µm C18 particle size, 100 Å pore size, 75 µm inner diameter, 25 cm length) at a flow rate of 300 nl/min at 60°C using a 2 h gradient from 4 to 40 % solvent B (0.1 % (v/v) formic acid, 84 % (v/v) acetonitrile in water) in solvent A (0.1 % (v/v) formic acid in water). The liquid chromatography system was online coupled to an Orbitrap Elite mass spectrometer via a nano electrospray ionization source and peptides injected by distal coated Silica Tip emitters using a spray voltage of 1.45 kV. The mass spectrometer was operated in positive, data-dependent mode with capillary temperature set to 225 °C. First, full scans (350-1700 m/z, resolution 60,000) were recorded in the

Orbitrap analyzer of the instrument with a maximal ion time of 200 ms and the target value for automatic gain control set to 1,000,000. In the linear ion trap part of the instrument subsequently up to twenty double- and triple-charged precursors with a minimal signal of 500 were isolated (isolation window 2 m/z), fragmented by collision induced dissociation (CID) and analyzed with a maximal ion time of 50 ms and the target value for automatic gain control set to 3000 (available mass range 50-2000 m/z, resolution 5400). Already analyzed precursors were excluded from further isolation and fragmentation for 45 sec.
For data analysis, the MaxQuant environment (version 1.5.3.8, was used with standard parameters if not otherwise stated. Spectra were searched against 20187 Swiss-Prot entries from the Homo sapiens proteome (UP000005640, downloaded on 18 th November 2015 from UniProt KB). Label-free quantification was enabled as well as the match between runs option. Tryptic cleavage specificity was chosen, as well as carbamidomethyl at cysteines as fixed and methionine oxidation, phosphorylation (threonine, serine and tyrosine), acetylation at protein n-termini and ubiquitination at lysine (GlyGly, +114.0429) as variable modifications. Mass tolerances were 20 ppm (first search) and 4.5 ppm (second search after recalibration) for precursor masses and 0.5 Da for fragment masses. Phosphorylation sites were reported showing the highest probability calculated form an MS/MS spectrum peak matches. Peptides and proteins were accepted at a false discovery rate of 1 %. For relative quantification of phosphorylated peptides, peptide intensities were normalized to progesterone receptor amounts by dividing them by the total progesterone receptor intensity.

Reverse Phase Protein Assay (RPPA)
The following primary antibodies were used (provider and product number): Akt (CST 4685), Akt-P-Ser473 (CST 4060), Akt-P-Thr308 (CST 9275), c-Fos (CST 4384), c-myc (CST 9402), c-myc-P-Thr58/Ser62 (Abcam ab32029), EGFR (ErB-1, HER1) (CST 4405), EGFR (ErB-1, HER1)-P-Tyr1068 (CST 2234), ER (estrogen receptor) (Thermo RM-9101-S), Erk1/2 (MAPK p44/42) (CST 4695), Erk1/2 (MAPK p44/42)-P-Thr202/Tyr204 (CST 4370), Her2 (Dako A0485), Her2-P-Tyr1221/Tyr1222 (CST 2243), MEK1 (CST 9124), MEK1/2-P-Ser217/Ser221 (CST 9154), MEK2 (CST 9125), Rb-P-Ser807/Ser811 (CST 8516), S6 ribosomal protein (CST 2217), S6 ribosomal protein-P-Ser235/Ser236 (CST 2211), S6 ribosomal protein-P-Ser240/Ser244 (CST 2215), PR (progesterone receptor) (sc-810).
The antibodies were purchased from CST (Cell Signaling Technology, Danvers, Massachusetts), Abcam (Abcam plc, Cambridge, UK), Thermo (Thermo Fisher Scientific, Waltham, Massachusetts), Dako (Dako Products, Hamburg, Germany), Santa Cruz (Santa Cruz Biotechnology, Dallas, USA).

Western Blot analysis and Immunofluorescence
The following primary antibodies were used (provider and product number): Akt (CST 4685), Akt-P-Ser473 (CST 4060), β-Actin (sc-2004), c-Fos (CST 4384), c-myc (CST 9402), CYP51A1 (ab210792), EGFR (ErB-1, HER1) (CST 4405), EGFR (ErB-1, HER1)-P-Tyr1068 (CST 2234), ERα (CST 8644), ERα (p-Ser118) (CST 2511), Erk1/2 (MAPK p44/42) (CST 4695), Erk1/2 (MAPK p44/42)-P-Thr202/Tyr204 (CST 4370), HER2 (CST 2165), Her2-P-Tyr1221/Tyr1222 (CST 2243), MEK1 (CST 9124), MEK1/2-P-Ser217/Ser221 (CST 9154), PGRMC1 (ab48012), PGRMC1 (13856), PR (progesterone receptor) (sc-810).
The antibodies were purchased from CST (Cell Signaling Technology, Danvers, Massachusetts), Abcam (Abcam plc, Cambridge, UK), Thermo (Thermo Fisher Scientific, Waltham, Massachusetts), and Santa Cruz (Santa Cruz Biotechnology, Dallas, USA).

qRT-PCR
We evaluated the qPCR results as follows: At first, we calculated the mean of all matching technical replicates $[C_T]$. Then we calculated ΔC_T with $\Delta C_T = C_{T\ Target\ Gene} - C_{T\ Reference\ Gene}$. Our reference was the expression of PDH, we subtracted its value from the other expression levels. Afterwards we determined $\Delta\Delta C_T$ with $\Delta\Delta C_T = \Delta C_{T\ Sample} - \Delta C_{T\ Reference\ Sample}$.
The standard deviation of ΔC_T is defined as $\sigma = \sqrt{\sigma^2_{Target\ Gene} + \sigma^2_{Reference\ Gene}}$ [with σ as the standard deviation]. At last the fold-difference, which is defined as the range: $[2^{-\Delta\Delta C_T+\sigma}; 2^{-\Delta\Delta C_T-\sigma}]$, was calculated.

Scatter Plots of breast cancer microarray data
The normalized data was obtained from the Gene Expression Omnibus (GEO, NCBI) and analysed via a R script. At first, we utilized GeoQuery for the GSE download. Afterwards we plotted the per sample expression values to ensure proper normalization. As the data contained technical replicates, we generated the mean value per sample. Finally, we calculated Spearman's correlation and plotted a scatterplot with a regression line in R.

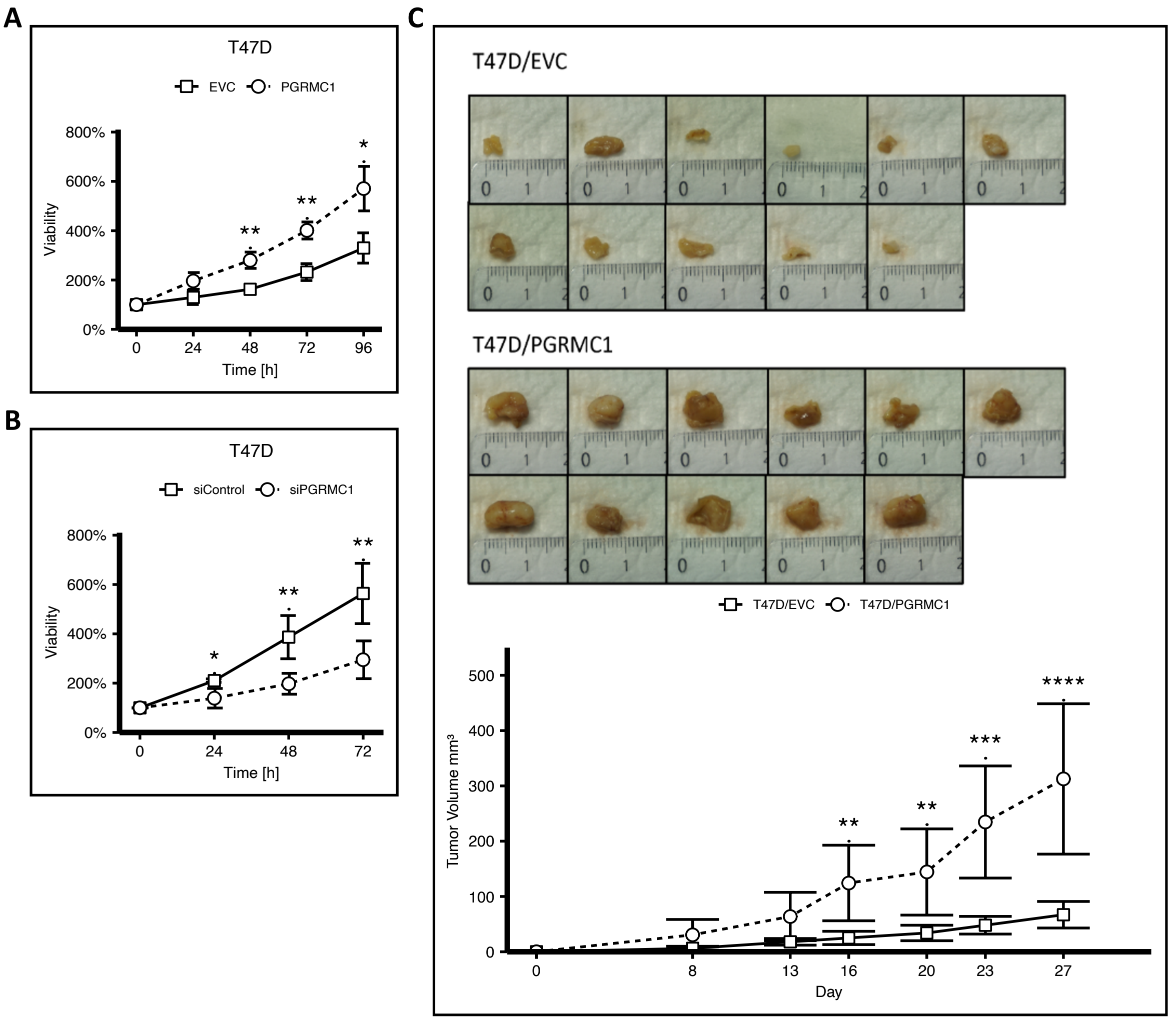

*Figure 1: (A) Cell viability of T47D/EVC and T47D/PGRMC1 cells (n = 3). Viability was analyzed by MTT assay at t = 0 h, 24h, 48h, 72h and 96h/37°C. Values were normalized to t = 0 (100%). *: p ≤ 0.05, **: p ≤ 0.01. (Student's t-test, n = 3). (B) Cell viability of T47D cells, treated with siRNA against PGRMC1 (siPGRMC1) and scrambled siRNA (siControl). (Student's t-test, n = 3). Viability was analyzed at t = 0h, 24h, 48h and 72h/37°C. Values were normalized to t = 0 (100%). *: p ≤ 0.05, **: p ≤ 0.01. (Student's t-test, n = 3). (C) Tumor volumes of immunodeficient mice bearing human breast cancer T47D/EVC and T47D/PGRMC1 xenografts. ***: p ≤ 0.001, ****: p ≤ 0.0001. (Student's t-test, n = 11 mice each group). Images of tumor tissue dissected from each mouse.*

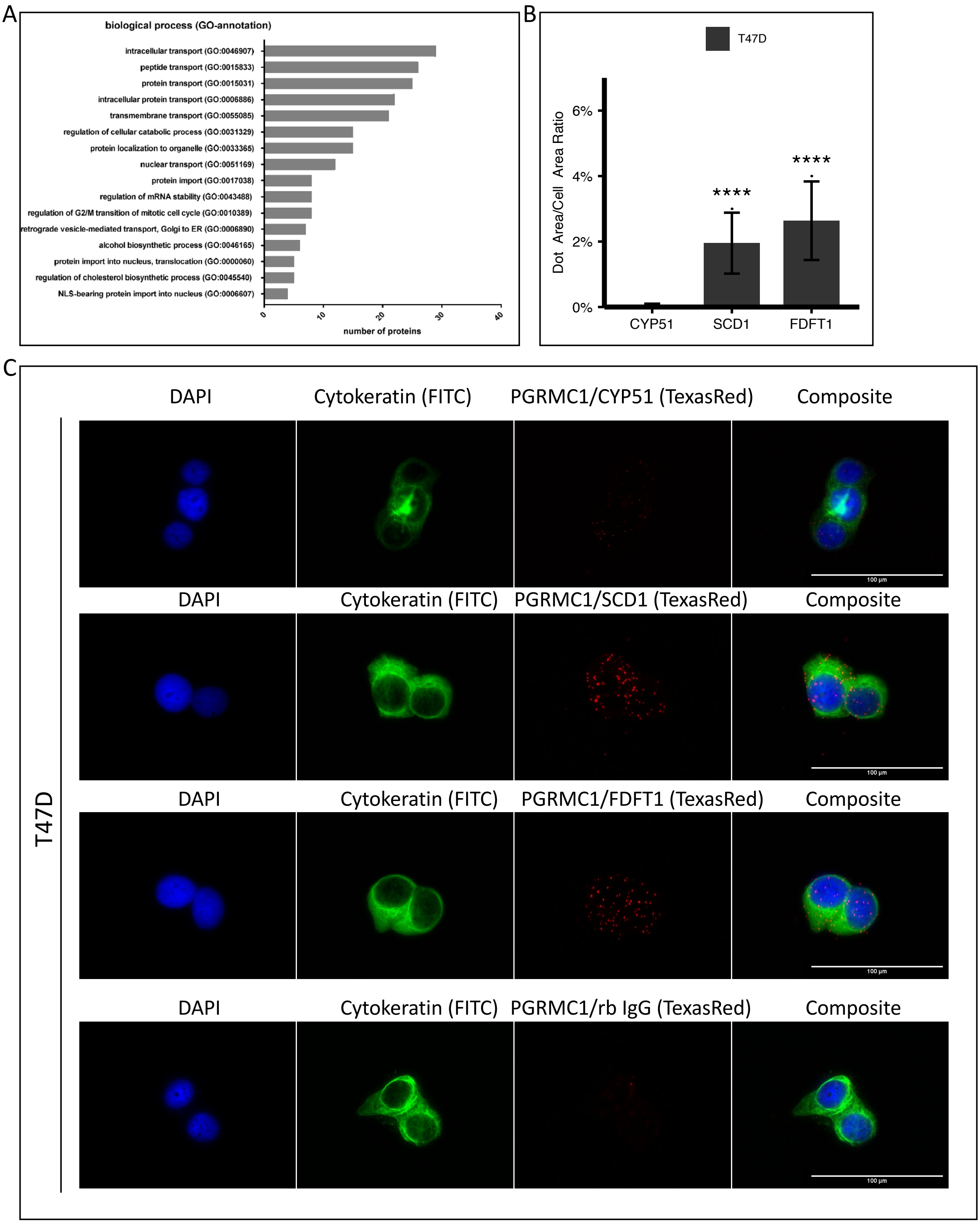
A
biological process (GO-annotation)
intracellular transport (GO:0046907)
peptide transport (GO:0015833)
protein transport (GO:0015031)
intracellular protein transport (GO:0006886)
transmembrane transport (GO:0055085)
regulation of cellular catabolic process (GO:0031329)
protein localization to organelle (GO:0033365)
nuclear transport (GO:0051169)
protein import (GO:0017038)
regulation of mRNA stability (GO:0043488)
regulation of G2/M transition of mitotic cell cycle (GO:0010389)
retrograde vesicle-mediated transport, Golgi to ER (GO:0006890)
alcohol biosynthetic process (GO:0046165)
protein import into nucleus, translocation (GO:0000060)
regulation of cholesterol biosynthetic process (GO:0045540)
NLS-bearing protein import into nucleus (GO:0006607)
0
10
20
30
40
number of proteins
B
T47D
Dot Area/Cell Area Ratio
6%
4%
2%
0%
****
****
CYP51
SCD1
FDFT1
C
DAPI
Cytokeratin (FITC)
PGRMC1/CYP51 (TexasRed)
Composite
DAPI
Cytokeratin (FITC)
PGRMC1/SCD1 (TexasRed)
Composite
DAPI
Cytokeratin (FITC)
PGRMC1/FDFT1 (TexasRed)
Composite
DAPI
Cytokeratin (FITC)
PGRMC1/rb IgG (TexasRed)
Composite
T47D

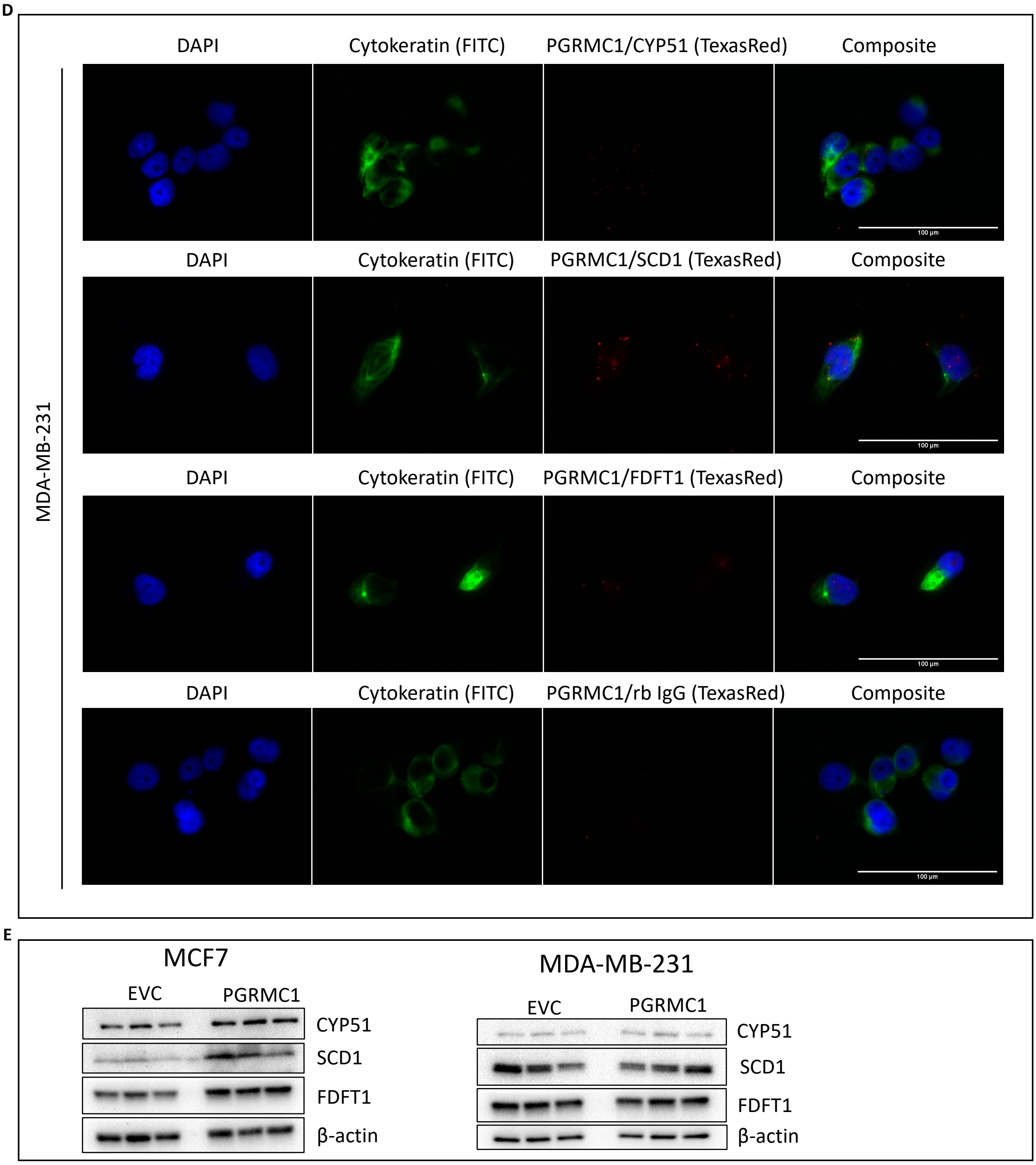

Figure 2: (A) PGRMC1 interaction partners associated with GO-annotations for biological functions. Number of proteins assigned to the respective biological function. The dataset was analyzed using Gene Ontology (geneontology.org). Total number of proteins in the dataset: 100. (B) PLA of PGRMC1 and Cyp51, FDFT1, SCD1 SCD1 and negative control rabbit isotype IgG (TexasRed) in T47D. Subsequent staining of Cytokeratin (FITC) and DAPI. Quantification of dots per cell and (C) Visualization via immunofluorescence microscopy. Magnification: 63 x. (D) PLA for protein interactions between PGRMC1 and CYP51, FDFT1, SCD1 and negative control rabbit isotype IgG (TexasRed) in MDA-MB-231 cells. Subsequent staining of Cytokeratin (FITC) and DAPI. Magnification: 63 x. (E) Western Blot analysis of CYP51, SCD1 and FDFT1 in MCF7/PGRMC1 cells and MDA-MB-231/PGRMC1 cells compared to their respective empty vector control.

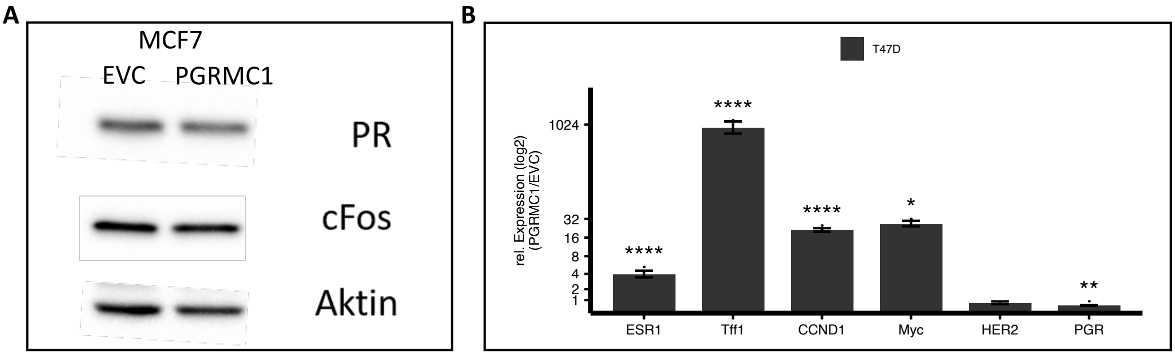

*Figure 3: (A) Western blot analysis of PR and cfos protein levels in MCF7/EVC and MCF7/PGRMC1 cells. (B) qRT-PCR analysis of ESR1, TFF1, HER2, CCND1, Myc and PR mRNA expression in T47D/EVC and T47D/PGRMC1 cells *: $p \leq 0.05$, ***: $p \leq 0.001$. (Student's t-test, n = 3)*

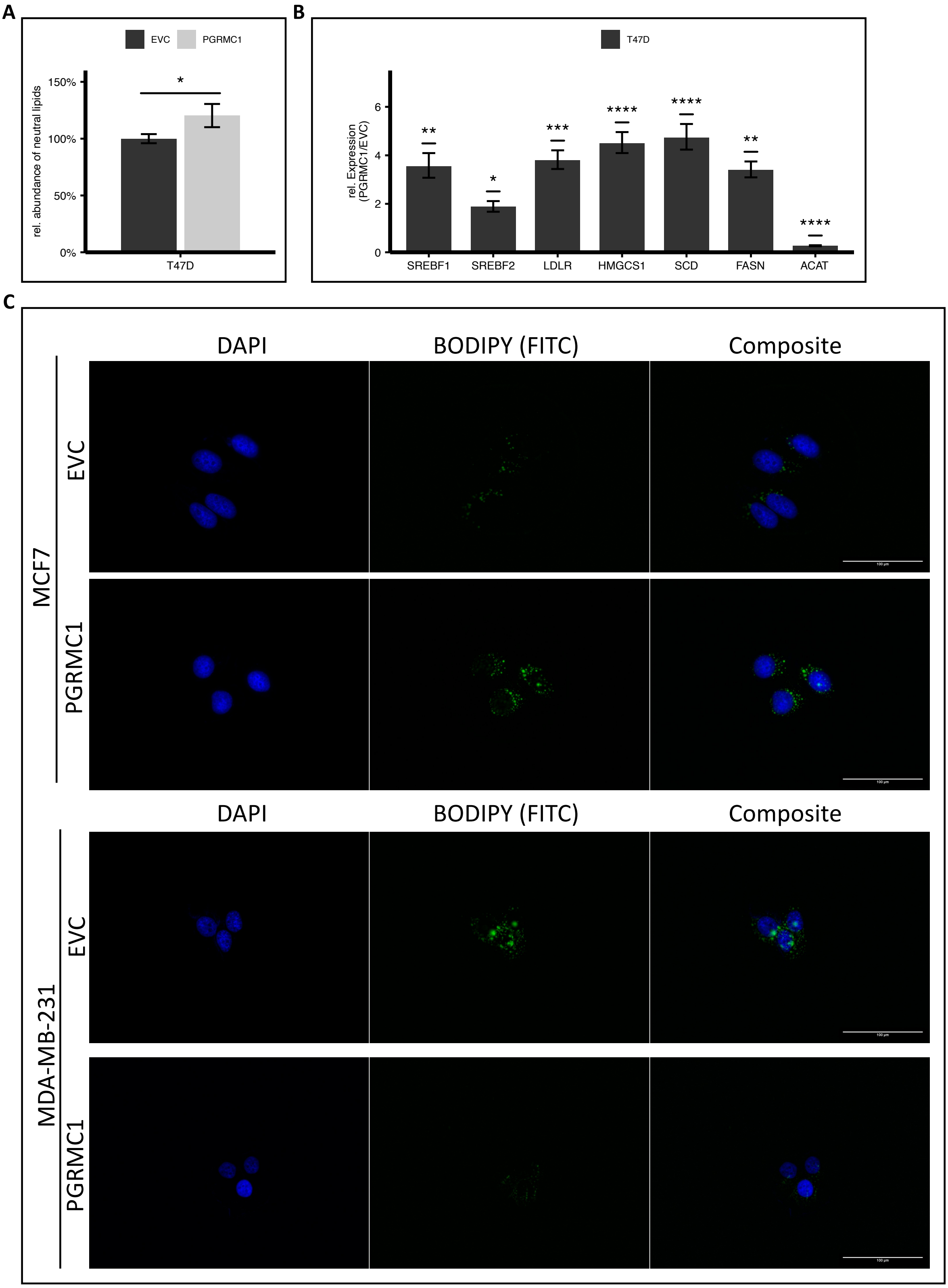
A
EVC
PGRMC1
rel. abundance of neutral lipids
150%
100%
50%
0%
*
T47D
B
T47D
rel. Expression (PGRMC1/EVC)
6
4
2
0
**
*
***
****
****
**
****
SREBF1
SREBF2
LDLR
HMGCS1
SCD
FASN
ACAT
C
DAPI
BODIPY (FITC)
Composite
MCF7
EVC
PGRMC1
DAPI
BODIPY (FITC)
Composite
MDA-MB-231
EVC
PGRMC1

D

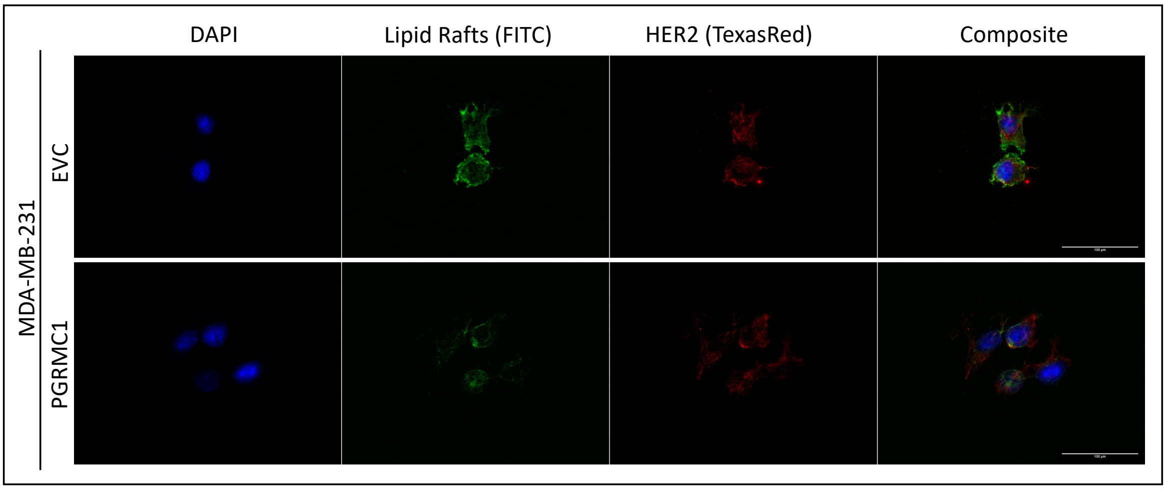

E

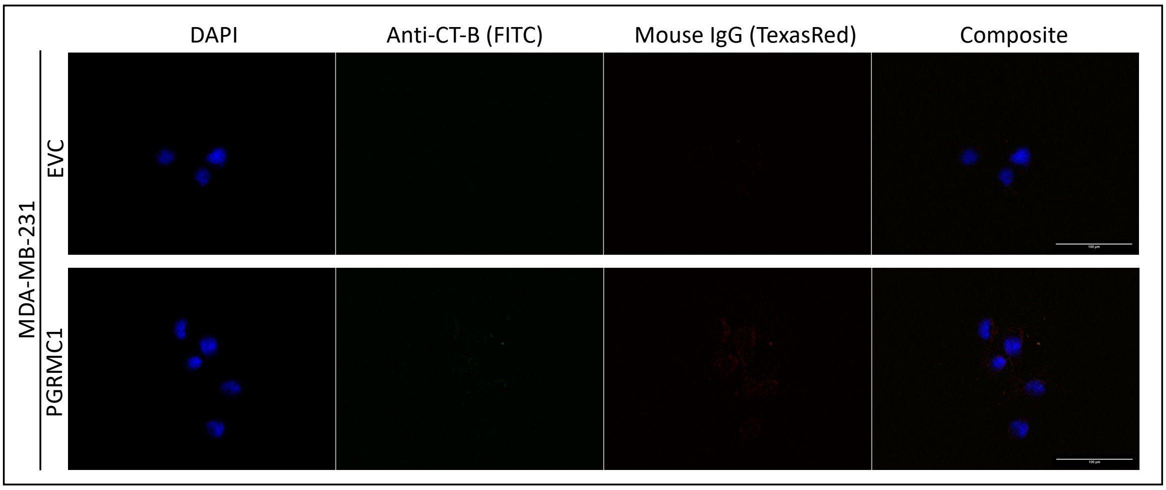

F

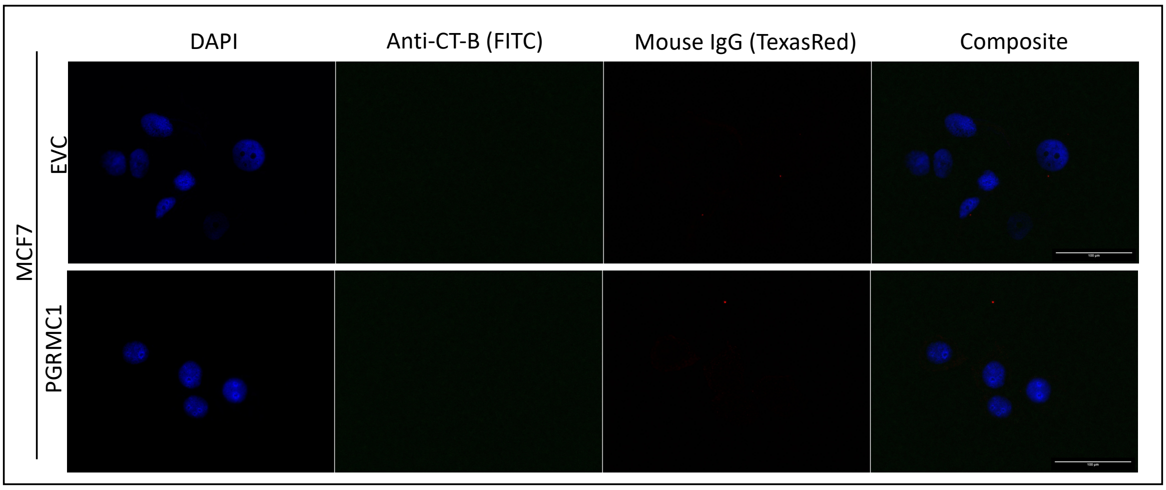

*Figure 4: (A) Detection of neutral lipids and lipid droplets in T47D/EVC and T47D/PGRMC1 cells by BODIPY® staining and quantification via flow cytometry. *: $p \leq 0.05$, **: $p \leq 0.01$. (Student's t-test, n = 3) (B) qRT-PCR analysis of SREPF1, SREBF2, LDLR, HMGS1, SCD, FASN, ACAT mRNA expression in T47D/PGRMC1 cells compared to the respective EVC cells. *: $p \leq 0.05$, **: $p \leq 0.01$, ***: $p \leq 0.001$. (Student's t-test, n = 3). (C) Staining of lipid droplets in MCF7/EVC and MCF7/PGRMC1, MDA-MB-231/EVC and MDA-MB-231/PGRMC1 with BODIPY™ 493/503. Nuclear stain with DAPI. Magnification: 63 x. (D) Immunofluorescence staining in MDA-MB-231/EVC and MDA-MB-231/PGRMC1 with Vybrant™ Alexa Fluor™ 488, fluorescence immunocytochemistry for HER2 and nuclear staining with DAPI. Magnification: 63 x. Negative control staining with rabbit isotype IgG (TexasRed) and Anti-CT-B only (FITC) in (E) MDA-MB-231/EVC and MDA-MB-231/PGRMC1 and (F) MCF7/EVC and MCF7/PGRMC1.*

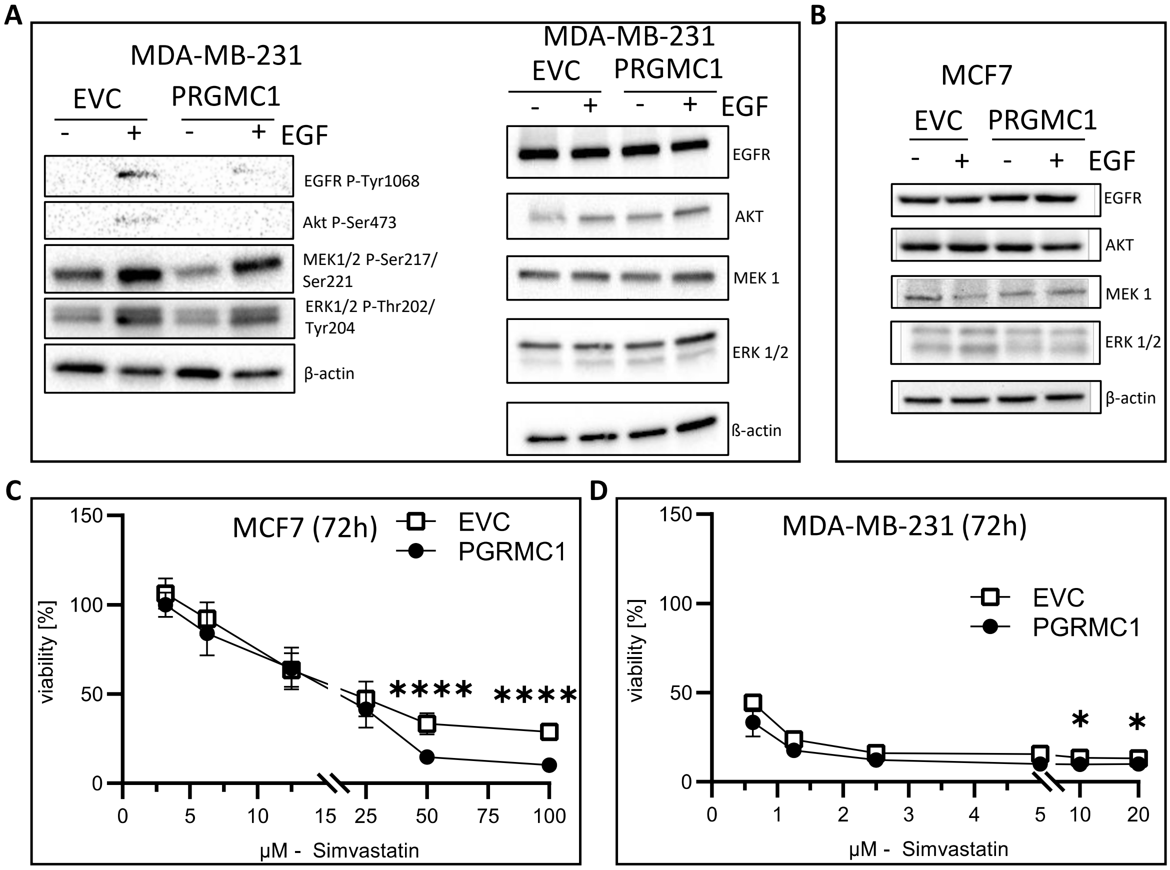

Figure 5 (A) Protein phosphorylation of EGFR P-Tyr1068, Akt P-Ser473, MEK1/2 P-Ser217/Ser221 and Erk1/2 P-Thr202/Tyr204) and total protein expression of EGFR, Akt, MEK1/2 and Erk1/2 in MDA-MB-231/EVC and MDA-MB-231/PGRMC1 analyzed by Western blot analysis. Cells were treated with EGF (10 ng/mL) for 10 min/37°C. (B) Total protein expression of EGFR, Akt, MEK1/2 and Erk1/2 in MCF7/EVC and MCF7/PGRMC1 analyzed by western blot analysis. MCF7/EVC and MCF7/PGRMC1 cells were treated with 100µM, 50µM, 25µM, 12.5µM, 6.25 µM and 3.175 µM simvastatin and respective DMSO control. MDA-MB-231/EVC and MDA-MB-231/PGRMC1 cells were treated with 20µM, 10µM, 5µM, 2.5µM, 1.25µm and 0.625µM simvastatin and respective DMSO control. Viability was analyzed by MTT assay at t=24h, t=48h, t=72h and 37°C. Depicted are results after 72 h (C, D) of treatment. Viability is normalized on the DMSO control. P-Values were adjusted using Bonferroni correction (n_{doses} = 6; $n_{replicates}$ = 9)
